# P-1444. Safety and Long-Term Immunogenicity of Yellow Fever Vaccine in Elderly Korean Travelers: A Retrospective Cohort Study

**DOI:** 10.1093/ofid/ofaf695.1630

**Published:** 2026-01-11

**Authors:** Jihyun Yang, Shinwon Lee, Sohee Park, Chae-Lim Song, Soon Ok Lee, Suhyeon Heo, Jeong Eun Lee, Kye-Hyung Kim, Sun Hee Lee

**Affiliations:** Korea Research Institute of Bioscience and Biotechnology, Daejeon, Taejon-jikhalsi, Republic of Korea; Division of Infectious Disease, Department of Internal Medicine, Pusan National University Hospital, Seo-gu, Pusan-jikhalsi, Republic of Korea; Pusan National University School of Medicine and Medical Research Institute, Pusan National University Hospital, Seo-gu, Pusan-jikhalsi, Republic of Korea; Pusan National University School of Medicine and Medical Research Institute, Pusan National University Hospital, Seo-gu, Pusan-jikhalsi, Republic of Korea; Division of Infectious Disease, Department of Internal Medicine, Pusan National University Hospital, Seo-gu, Pusan-jikhalsi, Republic of Korea; Korea Research Institute of Bioscience and Biotechnology, Daejeon, Taejon-jikhalsi, Republic of Korea; Division of Infectious Disease, Department of Internal Medicine, Pusan National University Hospital, Seo-gu, Pusan-jikhalsi, Republic of Korea; Pusan National University School of Medicine, Pusan, Pusan-jikhalsi, Republic of Korea; Division of Infectious Disease, Department of Internal Medicine, Pusan National University Hospital, Seo-gu, Pusan-jikhalsi, Republic of Korea

## Abstract

**Background:**

Yellow fever (YF) remains endemic in parts of Africa and South America. Although the YF vaccine, derived from the 17D strain, is highly effective, concerns remain regarding its safety and immunogenicity in elderly individuals over 60 years old. WHO classifies YF vaccination in this age group as a relative contraindication, recommending that healthcare providers carefully weigh the risks and benefits with recipients. As international travel from Korea to YF-endemic areas rises, including among older adults, data are needed to guide vaccination in this population.

**Figure 1.**
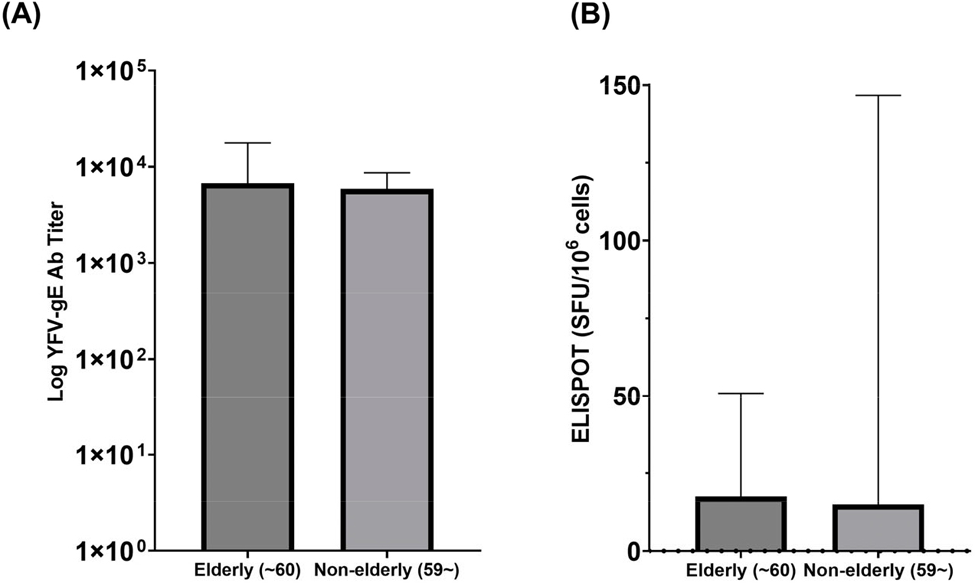
Comparison of (A) Humoral (YFV-gE Ab Titer) and (B) Cellular (IFN-γ ELISPOT) Immune Response Between in <60 and ≥60 age groups at a time point exceeding five years post-vaccination..

**Methods:**

We retrospectively reviewed adults vaccinated against YF at Pusan National University Hospital between September 2016 and January 2020. Adverse events (AEs) were assessed via telephone interviews. A subset of recipients underwent blood sampling for humoral and cellular immune response testing in 2024. Anti-YFV envelope IgG titers were measured by ELISA, and cellular responses were assessed via IFN-γ ELISPOT assays. Participants were grouped by age (< 60 vs. ≥60 years) for comparative analysis. A comparison of each group was performed using the Mann–Whitney Wilcoxon unpaired test and results were considered significant at P < 0.05.

**Results:**

Among 1,021 vaccinees, 691 (67.7%) were aged ≥60 years. No serious AEs were reported at the time of vaccination. Of the 454 individuals (44.5%) who completed telephone follow-up, 6 (1.3%) reported mild AEs (febrile sensation in 4; myalgia in 2) and no serious AE. Seventy-one individuals underwent immunogenicity assessment at a time point exceeding five years post-vaccination, with a median age of 62 years (49.3% male), including 44 individuals aged ≥60 years. Median log anti-YFV IgG titer was 3.83 (IQR, 3.55–4.25) in the elderly vs. 3.77 (IQR, 3.47–3.94) in the non-elderly (P = 0.55) (Figure 1). Median IFN-γ ELISPOT responses were 17.50 (IQR, 0–50.83) in the elderly vs. 15.00 (IQR, 1.67–146.7) in the non-elderly (P = 0.67) (Figure 1B).

**Conclusion:**

Yellow fever vaccination was well tolerated with no serious adverse events and preserved humoral and cellular immune responses in older adults. These findings support the safe use of YF vaccine in elderly Korean travelers, particularly when benefits outweigh risks in endemic-area travel.

**Disclosures:**

All Authors: No reported disclosures

